# Trans-Ned 19-Mediated Antagonism of Nicotinic Acid Adenine Nucleotide—Mediated Calcium Signaling Regulates Th17 Cell Plasticity in Mice

**DOI:** 10.3390/cells10113039

**Published:** 2021-11-05

**Authors:** Mikołaj Nawrocki, Niels Lory, Tanja Bedke, Friederike Stumme, Björn-Phillip Diercks, Andreas H. Guse, Chris Meier, Nicola Gagliani, Hans-Willi Mittrücker, Samuel Huber

**Affiliations:** 1 Section of Molecular Immunology and Gastroenterology, I. Department of Medicine, University Medical Center Hamburg-Eppendorf, 20246 Hamburg, Germany; m.nawrocki@uke.de (M.N.); t.bedke@uke.de (T.B.); f.stumme@uke.de (F.S.); n.gagliani@uke.de (N.G.); 2 Hamburg Center for Translational Immunology (HCTI), University Medical Center Hamburg-Eppendorf, 20246 Hamburg, Germany; n.lory@uke.de (N.L.); h.mittruecker@uke.de (H.-W.M.); 3 Institute of Immunology, University Medical Center Hamburg-Eppendorf, 20246 Hamburg, Germany; 4 The Calcium Signalling Group, Department of Biochemistry and Molecular Cell Biology, University Medical Center Hamburg-Eppendorf, 20246 Hamburg, Germany; b.diercks@uke.de (B.-P.D.); guse@uke.de (A.H.G.); 5 Institute of Organic Chemistry, Department of Chemistry, Faculty of Sciences, University of Hamburg, Martin-Luther-King-Platz 6, 20146 Hamburg, Germany; Chris.Meier@chemie.uni-hamburg.de; 6 Department of General, Visceral and Thoracic Surgery, University Medical Center Hamburg-Eppendorf, 20246 Hamburg, Germany; 7 Immunology and Allergy Unit, Department of Medicine Solna, Karolinska Institute, 17176 Stockholm, Sweden

**Keywords:** adenine nucleotides, NAADP, Ca^2+^ signaling, T cells, immune regulation, inflammatory diseases, immune therapy

## Abstract

Nicotinic acid adenine dinucleotide phosphate (NAADP) is the most potent Ca^2+^ mobilizing agent and its inhibition proved to inhibit T-cell activation. However, the impact of the NAADP signaling on CD4^+^ T-cell differentiation and plasticity and on the inflammation in tissues other than the central nervous system remains unclear. In this study, we used an antagonist of NAADP signaling, trans-Ned 19, to study the role of NAADP in CD4^+^ T-cell differentiation and effector function. Partial blockade of NAADP signaling in naïve CD4^+^ T cells in vitro promoted the differentiation of Th17 cells. Interestingly, trans-Ned 19 also promoted the production of IL-10, co-expression of LAG-3 and CD49b and increased the suppressive capacity of Th17 cells. Moreover, using an IL-17A fate mapping mouse model, we showed that NAADP inhibition promotes conversion of Th17 cells into regulatory T cells in vitro and in vivo. In line with the results, we found that inhibiting NAADP ameliorates disease in a mouse model of intestinal inflammation. Thus, these results reveal a novel function of NAADP in controlling the differentiation and plasticity of CD4^+^ T cells.

## 1. Introduction

Ca^2+^ in T cells controls key cellular processes: proliferation, differentiation, metabolism, cytokine-synthesis, and cytokine-secretion [1,2]. When a T cell is presented with cognate antigen by an antigen presenting cell, several Ca^2+^ mobilizing second messengers are produced: D-*myo*-inositol 1,4,5-triphosphate (IP_3_), cyclic ADP-ribose (cADPR) and nicotinic acid adenine dinucleotide phosphate (NAADP). They cause the release of Ca^2+^ from intracellular stores which increases the free cytoplasmic Ca^2+^ concentration ([Ca^2+^]_i_) and induces the activation of store-operated Ca^2+^ entry (SOCE) [1,2]. Influx of Ca^2+^ across the plasma membrane leads to a further [Ca^2+^]_i_ increase, which has been shown to play a critical role in T cell activation in multiple models of autoimmunity in vivo [3,4,5].

NAADP is the most potent Ca^2+^ mobilizing second messenger known to date, which was discovered initially as an impurity in nicotinamide dinucleotide phosphate (NADP) preparations [6]. Its mechanism of action and significance in T-cell biology have been an area of intensive research [2,7,8]. Thus, it has been shown that stimulation of Jurkat T cells with a CD3-specific antibody results in a biphasic increase of NAADP concentration, with a first peak occurring within 10 ms after activation and a second, long lasting, elevation after 5 min [9]. These results suggest that NAADP is involved in the early Ca^2+^ signaling. Indeed, microinjection of NAADP into T cells results in the formation of local Ca^2+^ signals which preceded the global increase of [Ca^2+^]_i_ [10]. Moreover, pharmacologic blockade and knock-down of ryanodine receptors (RYR) in Jurkat T cells significantly reduced NAADP induced Ca^2+^ release, suggesting that the ER located RYRs are the NAADP sensitive channels in the T lymphocytes [10,11]. Further supporting these findings, formation of T cell receptor (TCR) induced Ca^2+^ microdomains was markedly reduced or absent in primary murine *Ryr1^-/-^* T cells [12]. On the contrary, in pancreatic cells NAADP caused Ca^2+^ release from acidic stores via two pore channels (TPC) [13]. Interestingly, NAADP binding sites have not been identified on RYRs, nor on TPCs [14]. Recently, the hematological and neurological expressed 1-like protein (HN1L) was identified as an accessory molecule linking NAADP to RYR1 or TPC1 [15,16].

The significance of NAADP signaling in T-cell activation has been investigated with use of NAADP small molecule antagonists—BZ194 and trans-Ned 19 [17,18,19]. Both compounds were shown to specifically inhibit NAADP mediated Ca^2+^ release [17,18,19]. Furthermore, the blockade of NAADP signaling in primary T cells impaired global Ca^2+^ signals upon TCR ligation and downstream cellular processes such immunological synapse formation, proliferation and synthesis of cytokines [17,18,19]. Moreover, in vivo administration of BZ194 ameliorated the course of experimental autoimmune encephalomyelitis in rats [20]. The protective effect of NAADP inhibition has been attributed to the impaired T-cell activation and migration into the central nervous system [20]. Nevertheless, the role of NAADP in T-cell differentiation and in the induction of regulatory T cells has not yet been investigated.

T-cell differentiation is a process in which naïve CD4^+^ T cells integrate signaling from TCR stimulated by cognate antigen presentation and cytokine receptors activated by cytokines present in the specific tissue milieu [21]. Naïve CD4^+^ T cells can differentiate either into effector cells which factors coordinate the immune response or into regulatory cells which mediate the tolerance towards self or nonharmful antigens and prevent tissue damage from an overactivated immune response [21]. Differentiated effector CD4^+^ T cells are the orchestrators of the adaptive immune response. They secrete combinations of cytokines, which are adjusted to the type of offending agent [21]. Furthermore, differentiated CD4^+^ T cells exhibit a certain level of plasticity and can transdifferentiate into other subsets [22,23,24].

Although the function of NAADP has been thoroughly studied in Ca^2+^ signaling and activation of T cells, its role in the differentiation of primary naïve T cells has not been investigated. Moreover, the utility of NAADP blockade in vivo was investigated only in one preclinical rodent model. Therefore, this study aimed to thoroughly investigate the role of NAADP signaling in CD4^+^ T-cell differentiation and plasticity. Overall, we found that partial blockade NAADP signaling favored the differentiation of Th1 and Th17. Simultaneously it increased the production of IL-10 and co-expression of LAG-3 and CD49b by effector cells making these cells more suppressive. Moreover, it promoted the transdifferentiation of Th17 cells into regulatory T cells in vitro and in vivo. Finally, we found that inhibition of NAADP ameliorates disease in a mouse model of intestinal inflammation.

## 2. Materials and Methods

### 2.1. Mice

C57BL/6J and OT-II transgenic mice were obtained from the Jackson Laboratory. IL-10^eGFP^, IL-17A^eGFP^, IL-17A^FP635^, IFN-γ^FP635^, Foxp3^RFP^ reporter mice and Il17a^CRE^ and *Rosa26*^flox^STOP^flox^ eYFP have been described elsewhere [22,25,26,27]. Mice were kept under specific pathogen free conditions in the facility of the University Medical Center Hamburg-Eppendorf. Food and water were provided *ad libitum*. Age (7–18 weeks) and sex-matched littermates were used for all experiments. Animal procedures were approved by the review board of the City of Hamburg (Behörde für Soziales, Familie, Gesundheit und Verbraucherschutz, Hamburg, Germany, Registration numbers ORG_934 and 20/067). Both male and female mice were used in experiments. Animals were assigned randomly to experimental groups. Each cage contained animals of the different experimental groups.

### 2.2. Reagents

Fura2-AM was obtained from Life Technologies (Carlsbad, USA). The dye was dissolved in DMSO, divided into aliquots, and stored at −20 °C until required for use. Anti-mouse CD3 mAb (145-2C11), anti-mouse CD28 mAb (37.51), anti-mouse IFN-γ (XMG1.2), and anti-mouse IL-4 (11B11) were produced in house. Trans-Ned 19 was obtained from Tocris (Bremen, Germany) and BZ194 from Dept. of Organic Chemistry (University of Hamburg, Germany). BZ194 was synthesized according to the procedure reported in [17] All other chemicals were from Aldrich-Sigma-Merck (Darmstadt, Germany).

### 2.3. Cytokines

Murine IL-1β, IL-2, IL-6, IL-7, IL-12, IL-23, IL-27 were obtained from Miltenyi (Bergisch-Gladbach, Germany). Murine IL-4 and Human TGF-β1 were obtained from Biolegend (San Diego, CA, USA).

### 2.4. In Vivo Trans-Ned 19 Administration

Toxicity and pharmacokinetic parameters of trans-Ned 19 were reported elsewhere [28]. Adult male and female reporter and Fate mice were injected intraperitoneally 20 mg/kg of trans-Ned 19 dissolved in DMSO daily. Injected over a period of 1-week, trans-Ned 19 did not substantially affect weight of mice and it did not produce any adverse effects as monitored by postural and behavioral changes.

### 2.5. Anti-CD3 Antibody Treatment

To test the efficacy of trans-Ned 19 in intestinal inflammation, mice were injected with trans-Ned 19 in a dose 20 mg/kg dissolved in DMSO (Sigma Aldrich, St. Louis, MO, USA). Two hours after trans-Ned 19 administration, mice were injected with anti-CD3 mAb (145-2C11, 15 µg per mouse) intraperitoneally. Mice received a second dose of anti-CD3 mAb after 48 h. The mice were sacrificed 4 h after second injection.

### 2.6. Isolation of Cells from the Small Intestine of the Mouse

The small intestine was harvested with sterile instruments. After removal of Peyer’s patches, the small intestine was opened longitudinally and washed in PBS. The intestinal pieces were then incubated with 1mM Dithioeryhtritol (Applichem, Glenview, IL, USA) at 37 °C for 20 min followed by digestion with Clostridium Histolyticum collagenase (Sigma Aldrich, St. Louis, MO, USA) and DNase (BD Biosciences, Franklin Lakes, NJ, USA) at 37 °C for 45 min. The cells were further separated with a 67–40% Percoll gradient (GE Healthcare, Chicago, IL, USA).

### 2.7. Isolation of CD4^+^ T Cells from Murine Spleen and Lymph Nodes

Naïve or total CD4^+^ T cells were isolated from murine spleen and lymph node with Easysep^TM^ naïve CD4^+^ T cells or total CD4^+^T cells isolation kits (Stemcell, Vancouver, BC, Canada).

### 2.8. Imaging of Global Ca^2+^ Signalling in Primary T Cells from Mice

10^7^ freshly isolated naive murine CD4^+^ T cells were loaded with the membrane-permeable AM ester of Fura-2 (4 µM) for 15 min at 37 °C in 1 mL of full Click’s medium. After 15 min, 4 mL of fresh medium was added. The cells were rinsed twice and resuspended in Ca^2+^ measurement buffer (140 mM NaCl, 5 mM KCl, 1 mM MgSO_4_, 1 mM CaCl_2_, 20 mM Hepes (pH 7.4), 1 mM NaH_2_PO_4_, 5 mM glucose). The cells were then incubated with indicated concentrations of trans-Ned 19 or DMSO (0.1% *v/v*) at 37 °C for 30 min. Cells were added on prepared coverslips, coated with bovine serum albumin (5 mg/mL, Sigma-Aldrich) and poly-L-lysine (0.1 mg/mL, Sigma-Aldrich) and allowed to adhere before measurement.

Cells were stimulated by 2 µg/mL anti-CD3 antibody at the 20 s of recording Imaging was performed on a Leica IRBEmicroscope with 40x-fold magnification. A Sutter DG-4 was used as a light source, and frames were acquired with an electron-multiplying charge-coupled device camera (Hamamatsu). One frame every two seconds was acquired using a Fura-2 filter set (excitation, HC 340/26, HC387/11; beam splitter, 400DCLP; emission, 510/84; all in nanometers; AHF Analysentechnik). [Ca^2+^]_i_ was determined in Fura-2 loaded murine CD4^+^ T cells. Therefore, R_min_ [using the lowest ratio (R) and fluorescence (F) after EGTA chelation] and R_max_ [using the highest R and F after Ionomycin incubation] of Fura-2 in single-cell measurements were assessed.

### 2.9. Seahorse Metabolic Flux Measurement

CD4^+^ T cells were purified from spleens and lymph nodes of WT mice and incubated for 30 min with indicated concentration of trans-Ned 19 or DMSO and activated for 16 h with plate bound anti-CD3 antibody (10 µg/mL) and soluble anti-CD28 antibody (2 µg/mL). After activation, cells were harvested, washed twice with seahorse assay medium and transferred (10^5^/well) to poly-d-lysine precoated Seahorse microplates (Seahorse Bioscience, North Billerica, MA) plates were centrifuged to accumulate cells at the bottom of the plates. For determination of oxygen consumption rates (OCR), cells were washed and incubated in XF assay medium, 1 mM sodium pyruvate and 25 mM glucose (Seahorse Bioscience). Cells were analyzed using a XF-96 Extracellular Flux Analyzer (Seahorse Bioscience) according to the manufacturer’s protocols. During OCR analysis, cells were treated with 1.5 μM oligomycin, 1 µM fluorocarbonyl cyanide phenylhydrazone (FCCP), 1 μM rotenone and 1 μM antimycin A (Seahorse Bioscience). For the analysis of extracellular acidification rates (ECAR), cells were pretreated as described above and incubated in analysis plates in glucose- and pyruvate-free DMEM 5030 (Seahorse Bioscience). Cells were treated with 10 mM glucose, 1.5 μM oligomycin and 100 mM 2-DG (2-deoxyglucose, Seahorse Bioscience) during the assay. OCR and ECAR values were calculated using the program provided by the manufacturer.

### 2.10. Proliferation Assay

Naïve CD4^+^ T cells were loaded with 5 µM CellTrace violet dye (Thermofisher, Waltham, MA, USA) and activated by plate bound anti-CD3 antibody (2 µg/mL) in presence of soluble anti-CD28 antibody (1 µg/mL). Cells were incubated with increasing concentrations of trans-Ned 19 or DMSO (0.1%) for one hour prior to the activation. Proliferation was measured at day 2 of in vitro culture by assessing the dilution of the violet dye with flow cytometry.

### 2.11. Flow Cytometric Analysis

For surface staining, the cells were incubated with the following fluorochrome-conjugated monoclonal antibodies: anti-CD45 (clone: 30F11), anti-CD3 (clone: 17A2), anti-CD4 (clone: RM4-5), anti-CD8a (clone: 53-6.7), anti-CD49b (clone: Hma2), anti-CD69 (clone: H1.2F3), anti-LAG3 (clone: C9B7W), in the presence of a blocking anti-FcγR mAb (clone: 2.4G2) for 20 min at 4 °C. LAG-3 and CD49b were stained for 30 min at 37 °C. Unless otherwise specified, mAbs were purchased from Biolegend (San Diego, CA, USA).

To analyze intracellular cytokine production, purified cells were stimulated with 50 ng/mL phorbol 12-myristate 13-acetate (Sigma-Aldrich), 1 mM ionomycin (Sigma-Aldrich) and 2 µM monensin (Biolegend) for 4 h at 37 °C.

After 4 h, cells were stained with UV395-Zombie dye (Biolegend, San Diego, CA, USA) or with Pacific Orange Succinimydil Ester (Thermofisher, Waltham, MA, USA) to exclude dead cells. Afterwards, the cells were stained for cell surface markers as described above, then the cells were fixed with 4% formalin for 15 min and permeabilized with 0.1% NP-40 for 4 min and stained for 1 h. All steps were performed at room temperature. For intracellular staining, the following fluorochrome-conjugated monoclonal antibodies were used of anti-IFN-γ (clone: XMG1.2), anti-IL-17A (clone: TC11-18H10.1), anti-IL-10 (clone: JES5-16E3), anti-Nur77 (clone: 12.14), anti-IRF-4 (clone: 3E4). Cells were analyzed using a Fortessa flow cytometer (BD Biosciences, Franklin Lakes, NJ, USA) and FlowJo software (Tree Star, Ashland, OR, USA).

Trans-Ned 19 is a fluorescent molecule with excitation at 351 and 365 nm. We employed several strategies to compensate for the fluorescence of trans-Ned 19 and to prevent it from creating artifacts which would affect our results from flow cytometry. First, we did not use the UV laser and the BUV 395 channel in the acquisition of flow cytometry data (Supplementary Figure S1A,B). Nevertheless, the fluorescence of trans-Ned 19 had spectral overlap with the GFP channel which we used for the quantification of IL-17A and IL-10 expression (Supplementary Figure S1C,D). We report that the expression of these two cytokines is controlled by NAADP signaling, therefore it was for us of great importance to correctly analyze these results. To control for this spectral overlap, we employed the following gating strategy. We loaded the GFP negative cells with trans-Ned 19 in the same concentrations as we used for the tested conditions. We determined the cut off fluorescence intensity using these cells (Supplementary Figure S1E) to correctly gate the GFP positive cells (Supplementary Figure S1F).

### 2.12. In Vitro Differentiation of CD4^+^ T Cells

Lymphocytes were isolated from spleen and lymph nodes IL-17A^eGFP^ × IFN-γ^Katushka^ × Foxp3^RFP^ or IL-17A^Katushka^ × IL-10^eGFP^ × Foxp3^RFP^ reporter mice or Il17a^CRE^ × *Rosa26*^flox^STOP ^flox^YFP × IL-17A^Katushka^ × IL-10^eGFP^ × Foxp3^RFP^ fate tracking reporter or OT-II mice. Naïve CD4^+^CD25^-^CD44^-^ T cells were enriched by depletion of CD25^+^ and CD44^+^ cells followed by enrichment of CD4^+^ T cells using MACS according to the manufacturer’s instruction (Miltenyi Biotech, Bergisch-Gladbach, Germany). Purity of CD4^+^ T cells obtained was about 80% as determined by FACS. For each differentiation condition the cells were cultured in a 96-well plate at 2 × 10^5^ per well in 200 µL of full Click’s medium supplemented with the following cytokines and antibodies. For differentiation of Th1 cells, CD4^+^ naïve CD4^+^ T cells were cultured in the presence of 100 U/mL mIL-2, 10ng/mL mIL-12 and 10 µg/mL anti-IL4 mAb, and 2 µg/mL anti-CD28 mAb in plates coated with 10 µg/mL anti-CD3 mAb. For the differentiation of Th2 cells, naïve CD4^+^ T cells were cultured in the presence of 50 U/mL mIL-2, 20 ng/mL mIL-4 and 10 µg/mL anti-IFN-γ mAb, and 2 µg/mL anti-CD28 mAb in plates coated with 2 µg/mL anti-CD3 mAb. For the differentiation of Th17 cells, naïve CD4^+^ T cells were cultured in the presence of 10 ng/mL mIL-6 and 0.25 ng/mL hTGF-β1, 10 µg/mL anti-IL4 mAb, 10 µg/mL anti-IFN-γ mAb and 2 µg/mL anti-CD28 mAb in plates coated with 10 µg/mL anti-CD3 mAb. For differentiation of T regulatory cells, naïve CD4^+^ T cells were cultured in the presence of 50 U/mL mIL-2 and 2 ng/mL hTGF-β1 and 2 µg/mL anti-CD28 mAb in plates coated with 2 µg/mL anti-CD3 mAb. For differentiation of Tr1 cells, total CD4 ^+^ T cells were cultured in the presence of 30 ng/mL mIL-27 and 0.25 ng/mL hTGF-β1 and 2 µg/mL anti-CD28 mAb in plates coated with 10 µg/mL anti-CD3 mAb. In the experiments with trans-Ned 19, cells were incubated for one hour with increasing concentrations of trans-Ned 19 or DMSO (0.1%). In the experiments with BZ194, cells were incubated with increasing concentrations of BZ194 or DMSO (0.1%) for 5 h prior to stimulation.

### 2.13. In Vitro Suppression Assay

Responder cells were isolated from C57BL/6J mice and labelled with 5 µM CellTrace TM violet dye (Thermofisher, Waltham, USA). CD4^+^ T cells were isolated from IL-17A^eGFP^ × IFN-γ^FP635^ × Foxp3^RFP^ and cultured under Th17 polarizing conditions as described above in the presence of either 0.1% DMSO or 50 µM trans-Ned 19. IL-17A producing cells were then FACS sorted. 5 × 10^3^ responder cells were co-cultured with 12.5 × 10^3^ Th17 cells in presence of 2 × 10^5^ irradiated splenic feeder cells isolated from C57BL/6J mice. Proliferation of responder cells was analyzed at Day 4 of in vitro culture by assessing the dilution of the violet dye with flow cytometry.

### 2.14. In Vitro Cell Death Assay

CD4^+^ T cells isolated from C57BL/6J were cultured in a 96-well plate at 2 × 10^5^ per well in 200 µL of full Click’s medium supplemented with the 20 ng/mL of IL-7 and either DMSO (0.1%) or increasing concentrations of trans-Ned 19.

### 2.15. Statistical Analysis

Statistical analysis was performed with GraphPad Prism^®^ Software (GraphPad Software, San Diego, CA, USA). For comparison of 2 groups or multiple groups the non-parametric two-sided Mann–Whitney test or Kruskal-Wallis test was used, respectively. Bonferroni correction was used to counteract the problem in case of multiple comparisons. For analysis of in vitro experiments, the non-parametric paired Wilcoxon or Friedman test was used. The significance level alpha was set to 0.05.

For in vivo experiments the number of mice per group was calculated using an *a priori* power analysis for Wilcoxon-Mann-Whitney test to calculate the required sample size (G-power). The analysis was performed separately for each experiment and to calculate the power, we used data from our preliminary work.

## 3. Results

### 3.1. Antagonism of NAADP by Means of Trans-Ned 19 Inhibits Ca^2+^ Signaling upon TCR Stimulation, and Activation and Proliferation of CD4^+^ T Cells

**Figure 1:**
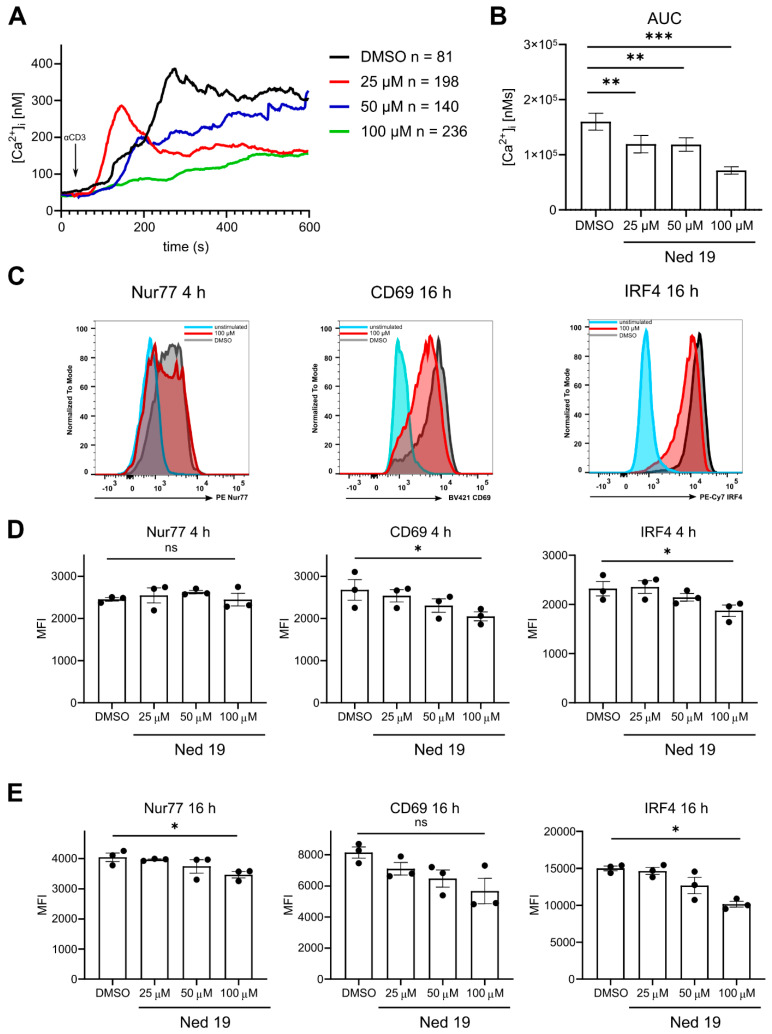
Trans-Ned 19 impairs Ca^2+^ and cell activation upon TCR stimulation. The naïve CD4^+^ T cells were freshly isolated from WT C57BL/6N mice, then incubated with increasing concentrations of trans-Ned 19 for 1 h. For Ca^2+^ signaling measurements, cells were loaded with Fura2-AM before incubation with trans-Ned 19. (**A**) Analysis of global Ca^2+^ measurements in naïve WT CD4^+^ T cells, showing mean Ca^2+^ traces upon soluble anti-CD3 mAb stimulation (2 µg/mL). (**B**) The area under the curve (AUC) of cytosolic [Ca^2+^]_i_ measured in the activated cells. (**C**) Representative flow cytometry histogram showing the expression of Nur77 at 4 h and CD69 and IRF4 at 16 h after activation. For these experiments, naïve CD4^+^ T cells were stimulated by plate bound anti-CD3 mAb (2 µg/mL) and soluble anti-CD28 mAb (1 µg/mL). (**D**) Pooled statistics of mean fluorescence intensity (MFI) of Nur77, CD69 and IRF4 measured 4 h after T-cell activation. (**E**) Pooled statistics of MFI of Nur77, CD69 and IRF4 measured 16 h after T-cell activation. Presented data are mean ± SEM. For (**B**), the *p*-values were calculated with ordinary one-way ANOVA with Dunnett’s multiple comparison test. For (**D**) and (**E**), *p*-values were calculated with Kruskal-Wallis multiple comparisons test. * *p* < 0.05, ** *p* < 0.01,*** *p* < 0.005.

**Figure 2:**
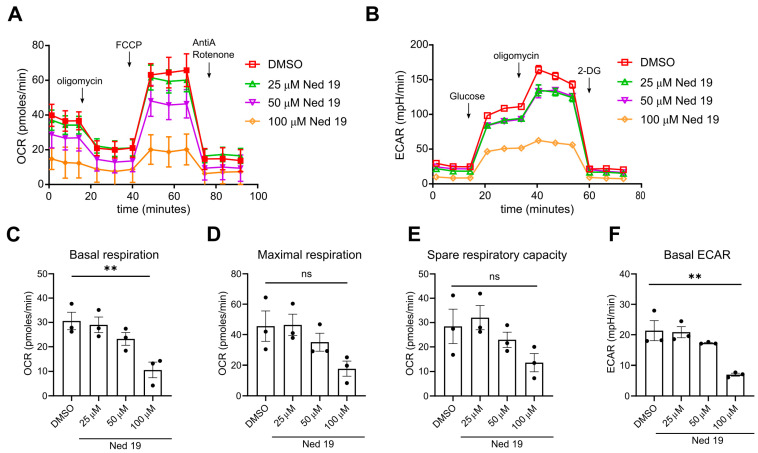
NAADP inhibition by means of trans-Ned 19 limits metabolic upregulation upon TCR stimulation in naïve CD4^+^ T cells. The naive CD4^+^ T cells were freshly isolated from triple reporter IL-17A^eGFP^ × IFN-γ^Katushka^ × Foxp3^RFP^ mice. The cells were incubated with increasing concentrations of trans-Ned 19 for 1 h, stimulated with plate bound anti-CD3 and soluble anti-CD28 mAbs, and cultured for 16 h in the presence of increasing concentrations of trans-Ned 19. (**A**) Representative oxygen consumption rate (OCR) measured by extracellular flux analysis. FCCP, carbonyl cyanide-p-trifluoromethoxyphenylhydrazone; AntiA, Rotenone + anti-mycin A. (**B**) Extracellular acidification rate (ECAR) measured by extracellular flux analysis. 2-DG—2-deoxyglucose. (**C**) Summary statistics of basal OCR. (**D**) Summary statistics of maximum OCR. (**E**) Summary statistics of spare respiratory capacity (**F**) Summary statistics of basal ECAR. Presented data are mean ± SEM. *p*-values were calculated with ordinary one-way ANOVA with Dunnett’s multiple comparison test. ** *p* < 0.01.

**Figure 3:**
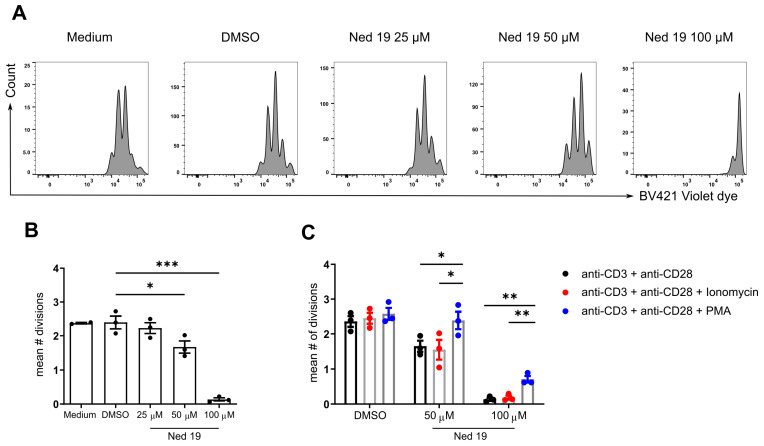
NAADP inhibition by means of trans-Ned 19 impairs naïve CD4^+^ T cell proliferation upon TCR stimulation which is reversible by PMA. The naïve CD4^+^ T cells were freshly isolated from WT C57BL/6N mice, loaded with violet dye, incubated with increasing concentrations of trans-Ned 19 for 1 h and stimulated with plate bound anti-CD3 mAb (2 µg/mL) and soluble anti-CD28 mAb (1 µg/mL). (**A**) Representative histograms of violet dye dilution. (**B**) Summary statistics of mean number of divisions indicated by violet dye dilution. (**C**) Summary statistics of proliferation assay with trans-Ned 19 and addition of either ionomycin (200 ng/mL) or PMA (100 ng/mL). Presented data are mean ± SEM. The *p*-values were calculated with repeated measures ANOVA with Dunnett’s multiple comparison test. * *p* < 0.05, ** *p* < 0.01, *** *p* < 0.005.

It was shown previously that NAADP plays a pivotal role in early TCR induced Ca^2+^ signaling and the small antagonists of NAADP impair TCR ligation mediated [Ca^2+^]_i_ increase and consequently inhibit Ca^2+^ dependent cellular activation events [17,18]. To confirm these findings and to validate the effect of trans-Ned19, we incubated Fura-2 loaded murine naïve CD4^+^ T cells with trans-Ned 19 and activated them with soluble anti-CD3 mAb. We observed a concentration-dependent decrease of Ca^2+^ signaling upon anti-CD3 mAb stimulation, albeit less profound as reported by Ali et al. [18] (Figure 1A,B). Additionally, we investigated the impact of NAADP inhibition by trans-Ned 19 on T-cell activation. To this end, naïve CD4 T cells were stimulated in vitro with anti-CD3 and anti-CD28 antibodies and expression of activation markers Nur77, CD69 and IRF4 was assessed via flow cytometry. Among these, Nur77 and IRF4 specifically reflect the TCR signal strength [29,30]. Indeed, increasing the concentration of trans-Ned 19 correlated with the decreased activation status 4 and 16 h following TCR stimulation (Figure 1C–E). In the next step of activation, naïve T cells undergo metabolic reprogramming in order to meet the increased energy expenditure required for cytokine synthesis [31]. We performed metabolic flux assays to evaluate the effects of NAADP antagonism on these processes. Mitochondrial stress tests and glycolysis stress tests indicated that trans-Ned 19 impairs the process of TCR induced metabolic up-regulation regarding both mitochondrial respiration and glycolysis in a concentration-dependent manner (Figure 2). Moreover, we evaluated the effect of trans-Ned 19 on naïve CD4^+^ T-cell proliferation following anti-CD3 mAb stimulation and showed that trans-Ned 19 inhibits the proliferation of naïve CD4^+^ T cells (Figure 3A,B).

To further dissect the NAADP signaling in the TCR/CD3 activation cascade, we used a Ca^2+^ ionophore ionomycin and protein kinase C activator phorbol-12-myristat-13-acetate (PMA) and investigated whether they can reverse trans-Ned 19 induced inhibition of proliferation. Surprisingly, elevating global [Ca^2+^]_i_ with ionomycin did not reverse the effect of trans-Ned 19 while PMA was able to reverse the inhibition of proliferation at concentration of 50 µM of trans-Ned 19 and partially restored the complete block of T-cell proliferation induced by treatment with 100 µM of trans-Ned 19 (Figure 3C).

One obvious concern regarding the pharmacological probes is their specificity. Specific blocking of NAADP induced Ca^2+^ release by trans-Ned 19 and no disruption of IP_3_ or cADPR induced Ca^2+^ release was reported by Naylor et al. [19]. Moreover, to exclude the nonspecific toxic effects of trans-Ned 19, we incubated CD4^+^ T cells with increasing concentration of trans-Ned 19 for 72 h in vitro without TCR/CD3 stimulation. In this setting, concentrations of trans-Ned 19 up to 50 µM did not significantly impair cell viability (Supplementary Figure S2).

Taken together, NAADP plays an important role in mediating TCR induced Ca^2+^ signals and naïve CD4^+^ T-cell activation and proliferation. Moreover, we have shown that trans-Ned 19 inhibits T-cell proliferation upon TCR stimulation which can be reversed by PMA but not ionomycin and that these effects are not due to unspecific toxic effects of the compound.

### 3.2. NAADP Inhibition Promotes the Differentiation of Th1 and Th17 Cells and It Inhibits the Differentiation of Foxp3^+^ Regulatory T Cells In Vitro

Having shown that NAADP signaling plays a pivotal role in the TCR activation of CD4^+^ T cells, we proceeded to investigate the role of NAADP in the differentiation of CD4^+^ T cells. Ali et al. has reported that trans-Ned 19 inhibits the synthesis of IL-2, IFN-γ, IL-4 and IL-17A. However, in this report the concentration of trans-Ned 19 used was 100 µM which resulted in a complete inhibition of T-cell proliferation. Therefore, we investigated the effects of partial blockade of NAADP signaling on CD4^+^ T-cell differentiation.

**Figure 4:**
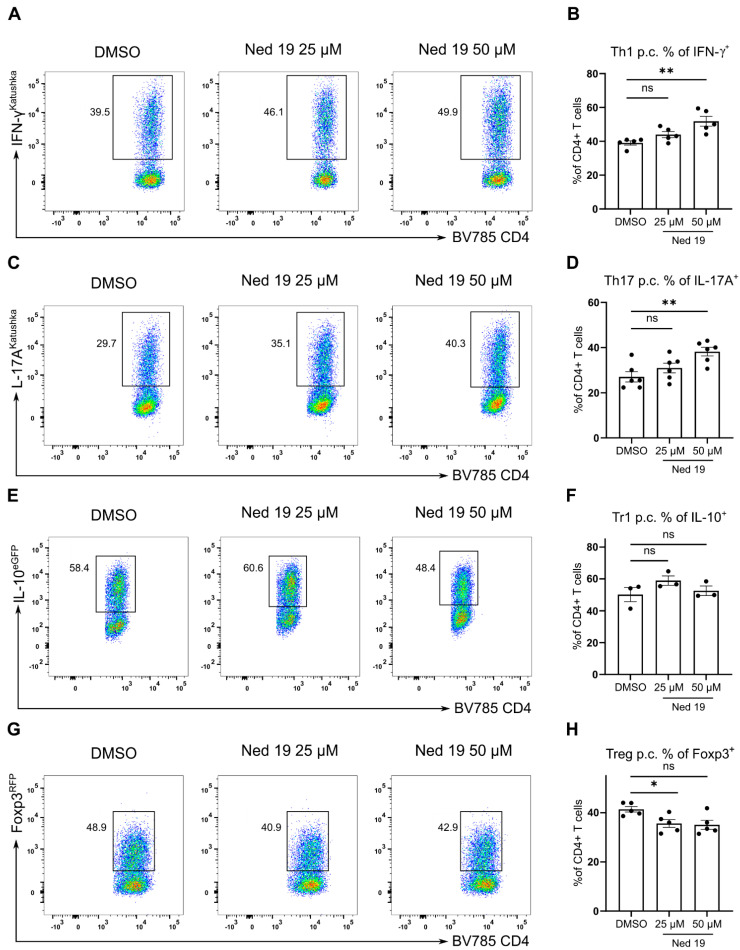
Trans-Ned 19 promotes the differentiation of Th1 and Th17 cells without a significant impact on the differentiation of regulatory T cells in vitro. Total CD4^+^ T cells were freshly isolated from IL-17A^Katushka^ × IL-10^eGFP^ × Foxp3^RFP^ mice for Tr1 in vitro differentiation and naïve CD4^+^ T cells from IL-17A^eGFP^ × IFN-γ^Katushka^ × Foxp3^RFP^ for Th1, Th17 and Treg cells. Cells were incubated with increasing concentrations of trans-Ned 19 for 1 h and stimulated with plate bound anti-CD3 mAb, soluble anti-CD28 mAb and polarizing cytokine cocktails. (**A**) Representative dot plots of in vitro Th1-cell differentiation. Percent of IFN-γ^+^ cells of CD4^+^ T cells is displayed. (**B**) Summary statistics of Th1-cell differentiation (p.c. polarizing conditions). (**C**) Representative dot plots of in vitro Th17-cell differentiation. Percent of IL-17A^+^ cells of CD4^+^ T cells is displayed. (**D**) Summary statistics of Th17-cell differentiation. (**E**) Representative dot plots of in vitro Tr1-cell differentiation. Percent of IL-10^+^ cells of CD4^+^ T cells is displayed. (**F**) Summary statistics of Tr1 differentiation. (**G**) Representative dot plots of in vitro Treg-cell differentiation. Percent of Foxp3^+^ cells of CD4^+^ T cells is displayed. (**H**) Summary statistics of Treg-cell differentiation. Presented data in (**B**,**D**,**F**,**H**) are mean ± SEM. The *p*-values were calculated with repeated measures ANOVA with Dunnett’s multiple comparison test. * *p* < 0.05, ** *p* < 0.01.

To this end, we used the naïve CD4^+^ T cells isolated from triple reporter mice (IL-17A^eGFP^ × IFN-γ^Katushka^ × Foxp3^RFP^ or IL-17A^Katushka^ × IL-10^eGFP^ × Foxp3^RFP^) which indicate cytokine or Foxp3 synthesis with expression of fluorescent proteins. Naïve CD4^+^ T cells were incubated with increasing concentrations of trans-Ned 19 and stimulated with anti-CD3 and anti-CD28 antibodies in the presence of polarizing cytokine cocktails. Unexpectedly, increasing concentrations of trans-Ned 19 promoted differentiation of IFN-γ producing cells under Th1 polarizing conditions, and IL-17A production under Th17 polarizing conditions, while impairing the differentiation of induced Foxp3^+^ Tregs (iTregs). Trans-Ned 19 had no impact on the differentiation of IL-10 producing cells under Tr1 polarizing conditions (Figure 4). To confirm these results after stimulation with cognate antigen, we reproduced the in vitro experiments regarding Th1 and Th17-cell differentiation with naïve CD4^+^ T cells isolated from OT-II mice stimulated with ovalbumin peptide loaded antigen presenting cells (Supplementary Figure S3).

The specificity of small molecule inhibitors might pose an impediment to the interpretation of these results. Therefore, we used another well-defined NAADP antagonist BZ194 [17] and performed analogous in vitro differentiation assays. The experiments revealed that the inhibition of NAADP signaling with BZ194 mirrored the results of trans-Ned 19 regarding Th1 and Th17 cell differentiation (Supplementary Figure S4A,B). Thus, arguing that the observed effects are the result of NAADP antagonism and not substance specific off-target effect. Nevertheless, the required concentrations of BZ194 were 10 times higher than in case of trans-Ned 19, which is in line with previous reports using BZ194 [17]. Of note, studies which administered BZ194 in vivo reported a significant toxicity, therefore, we did not perform in vivo experiments with BZ194 [32].

In summary, incomplete blockade of NAADP signaling in naïve CD4^+^ T cells promotes development of Th1 and Th17 effector CD4^+^ T cells.

### 3.3. NAADP Inhibition with Trans-Ned 19 Promotes Production of IL-10 by In Vitro Differentiated Effector T Cells and Foxp3^+^ T Regulatory Cells and Increases Their Suppressive Capacity

In previous reports, NAADP antagonist BZ194 ameliorated the course of autoinflammatory conditions [20,33]. Moreover, studies on the role of Ca^2+^ signaling and T-cell differentiation revealed that impaired TCR induced Ca^2+^ signaling is associated with the diminished cytokine production and the development of the effector CD4^+^ T cells [3,4,5]. Hence, the finding that NAADP inhibition by means of trans-Ned 19 promoted the differentiation of effector cells producing pro-inflammatory cytokines and inhibited development of Tregs in vitro was unexpected. Therefore, we sought to further characterize the in vitro differentiated cells qualitatively for the markers of possible suppressive capacity.

**Figure 5:**
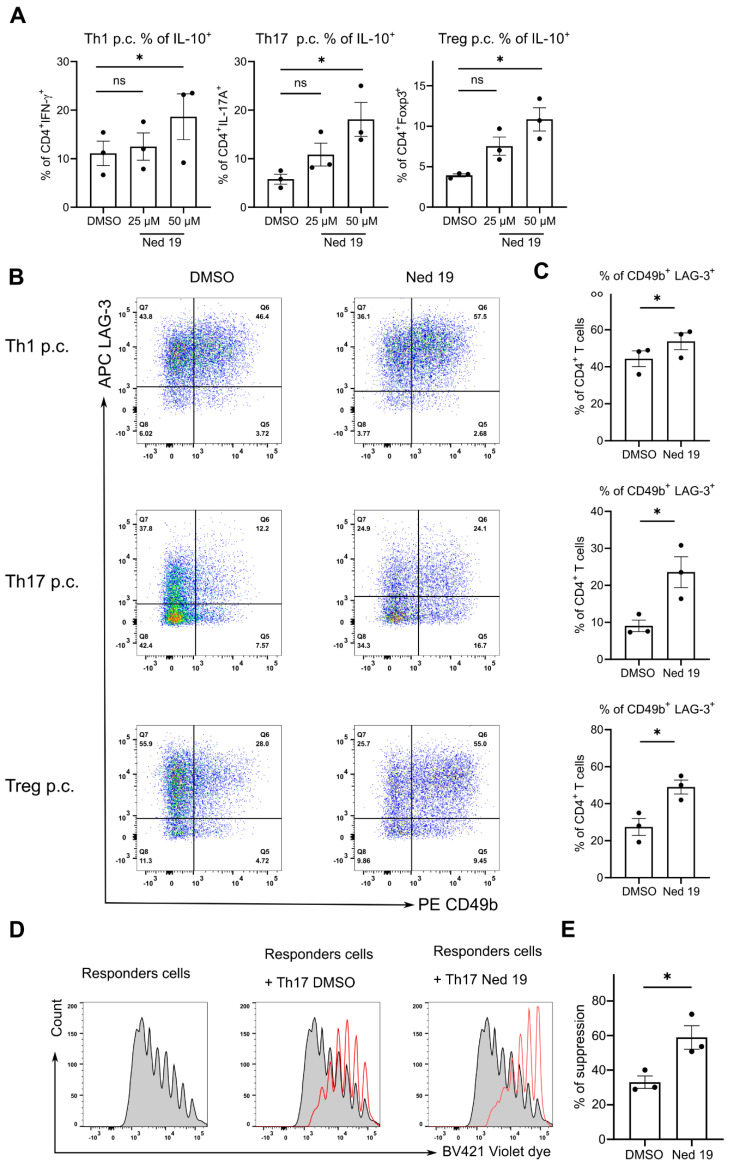
Trans-Ned 19 promotes the expression of IL-10, LAG-3 and CD49b by Th1, Th17 and Tregs cells in vitro and promotes the suppressive capacity of Th17 cells in vitro. The naive CD4^+^ T cells were freshly isolated from IL-17A^Katushka^ × IL-10^eGFP^ × Foxp3^RFP^ triple reporter mice. The cells were incubated with increasing concentrations of trans-Ned 19 for 1 h or DMSO (0.1%), stimulated with plate bound anti-CD3 mAb, soluble anti-CD28 mAb and polarizing cytokine cocktails, and cultured for 96 h in the presence of trans-Ned 19 or DMSO (0.1%). (**A**) Summary statistics for IL-10 expression under Th1, Th17 and Treg polarizing conditions (p.c.). (**B**) Representative dot plots of CD49b and LAG-3 expression on in vitro polarized Th1, Th17 and Treg cells in the presence of DMSO (0.1%) or trans-Ned 19 (50µM) (**C**) Summary statistics of CD49b^+^LAG-3^+^ cells among CD4^+^ T cell cultured under Th1, Th17 and Treg cell polarizing conditions (p.c.). (**D**) Representative histograms showing loss of violet dye as indication of proliferation (grey: responder cells without Th17 cells, red: with Th17 cells generated with and without trans-Ned 19. (**E**) Summary statistics of suppression by Th17 cells polarized in the presence or absence of trans-Ned 19. Percent of suppression was calculated as proliferation index normalized to the control proliferation index in the absence of suppressive cells. Presented data in B and D are mean ± SEM. The *p*-values were calculated with paired student’s Wilcoxon test. * *p* < 0.05.

In the next step, we evaluated the expression of IL-10, a canonical immunosuppressive cytokine, by the differentiated T cells. Interestingly, trans-Ned 19 promoted the production of IL-10 by in vitro differentiated Th1, Th17 and Foxp3^+^ Treg cells (Figure 5A). Brockmann et al. reported that highly suppressive IL-10 producing cells can be identified by expression of CD49b and the co-inhibitory receptor LAG-3 [34]. Therefore, we investigated how the expression of these proteins was affected by treatment with Trans-Ned 19. Interestingly, in line with promoting IL-10 production, treatment with trans-Ned 19 increased the proportion of double positive CD49b^+^ LAG-3^+^ cells in Th1, Th17 and Treg cells (Figure 5B,C). We noted the most profound induction of CD49b and LAG-3 in Th17 cells and therefore tested whether trans-Ned 19 treatment affected their suppressive capacity. To this end, we measured the proliferation of naïve CD4^+^ T cells co-cultured with Th17 cells differentiated either in the presence of DMSO or trans-Ned 19. In line with the increased expression of IL-10 and greater proportion of CD49b^+^LAG-3^+^ double positive cells, blocking NAADP pathway promoted the suppressive capacity of Th17 cells (Figure 5D,E).

### 3.4. NAADP Inhibition Promotes the Transdifferentiation of Th17 Cells into T Regulatory Type 1 Cells In Vitro and In Vivo

**Figure 6:**
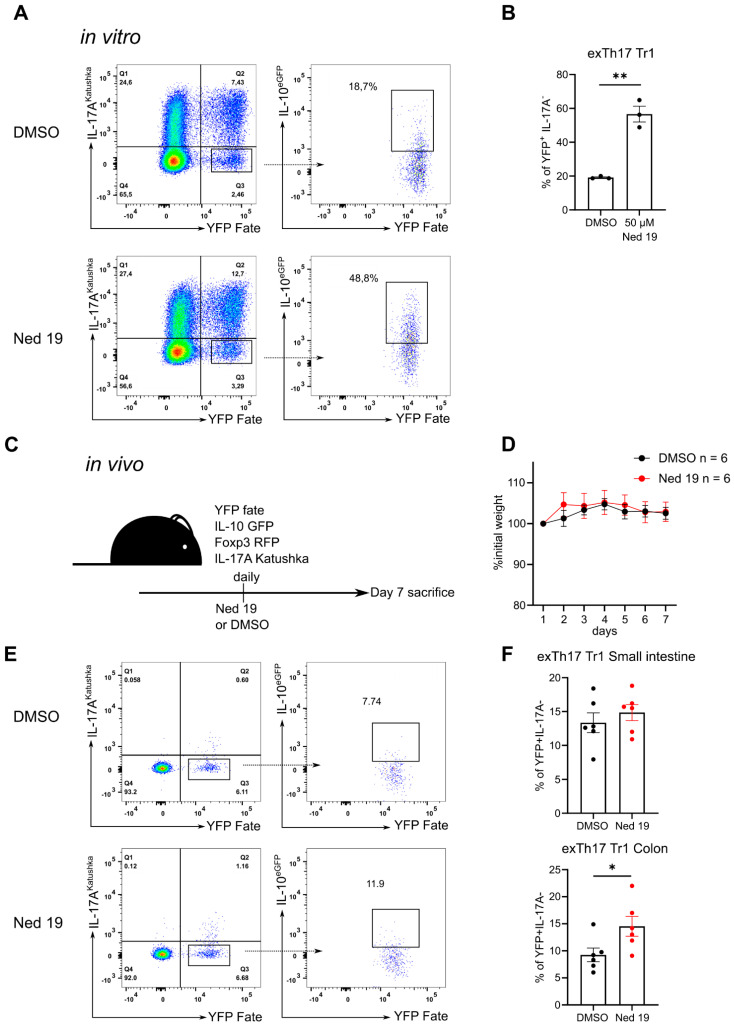
NAADP inhibition by trans-Ned 19 promotes the trans-differentiation of Th17 cells into Tr1 cells in vitro and in vivo. Naive CD4^+^ T cells were freshly isolated from Fate reporter mice (IL-17A^Cre^ × Rosa26 STOP^fl/fl^ YFP × IL-17A^Katushka^ × IL-10^eGFP^ × Foxp3^RFP^ mice). The cells were incubated with increasing concentrations of trans-Ned 19 for 1 h, stimulated with plate bound anti-CD3 mAb, soluble anti-CD28 mAb and cultured for 96 h in the presence of trans-Ned 19 under Th17 polarizing conditions. (**A**) Representative dot plots of cells cultured under Th17 polarizing conditions. Gating strategy for exTh17 cells (IL-17A^-^ IL17-fate/YFP^+^) on the left. Analysis of IL-10 expression by exTh17 Tr1 cells on the right (**B**) Summary statistics of IL-10^+^ IL-17A^-^ IL17-fate/YFP^+^ exTh17 Tr1-cell differentiation in vitro. (**C**) Experimental scheme of in vivo trans-Ned 19 administration. (**D**) Percentage of initial body weight upon administration of DMSO or trans-Ned 19. (**E**) Representative dot plots of colonic CD4^+^ T cells isolated from Fate reporter mice upon administration of trans-Ned 19 or vehicle control. (**F**) Summary statistics of IL-10^+^ IL-17A^-^ IL17-fate/YFP^+^ exTh17 Tr1-cell isolated from small intestine and colon of Fate reporter mice upon administration of trans-Ned 19 or vehicle control. Presented data in (**B**), (**D**), and (**F**) are mean ± SEM. The *p*-values were calculated with Mann-Whitney test. * *p* < 0.05, ** *p* < 0.01.

CD4^+^ T cells and especially Th17 cells exhibit a certain level of plasticity, as they can downregulate the expression of IL-17A and start expressing high levels of IFN-γ or IL-10 [22,24]. As mentioned above, NAADP inhibition promotes production of IL-10 by effector T cells and Foxp3^+^ Treg cells, however, it did not have an impact on the differentiation of IL-10 producing Tr1 cells. This suggests that NAADP controls the plasticity of T cells by restraining the conversion of effector cells into IL-10 producing suppressive cells. To test this hypothesis, we made use of the IL-17A fate mapping mouse model. In this mouse model, cells which express high levels of IL-17A delete the stop cassette preceding the *Yfp* gene and are permanently marked by *Yfp* expression [22]. Moreover, this mouse line is transgenic for fluorescent reporter proteins indicating the expression of *Il17a*, *Il10* and *Foxp3* genes [22]. We used the naïve CD4^+^ T cells from the Fate reporter mouse and differentiated them into Th17 cells in the presence of trans-Ned 19, which revealed that trans-Ned 19 significantly promotes the plasticity of Th17 cells towards conversion into exTh17 Tr1 cells identified by the IL-17A fate^+^ IL-17A^-^ IL-10^+^ Foxp3^-^ phenotype (Figure 6A,B). We confirmed these results using a second well defined NAADP antagonist BZ194 (Supplementary Figure S4C). In the next step, we evaluated whether this phenomenon occurs in the in vivo setting. To this end, Fate reporter mice were administered trans-Ned 19 for 7 days. Intestinal lamina propria is a site where T cells are constantly presented bacterial antigens and therefore, Th17, Th1 and T regulatory cells are present in high frequencies in steady state [35]. Trans-Ned 19 administration had no major impact on the mouse weight (Figure 6D). Trans-Ned 19 treated mice had a higher proportion of IL-17A fate^+^ IL-17A^-^ IL-10^+^ CD4^+^ T cells in the lamina propria of the small intestinal and colonic mucosa (Figure 6E,F). Thus, trans-Ned 19 also promoted the development of exTh17 Tr1 cells in vivo.

### 3.5. NAADP Inhibition In Vivo Ameliorates Disease in the Anti-CD3 Induced Intestinal Inflammation Mouse Model

**Figure 7:**
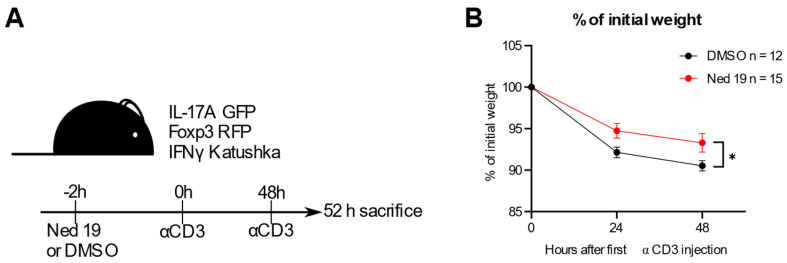
NAADP inhibition by trans-Ned 19 ameliorates course of disease in the anti-CD3 mAb induced duodenitis model. (**A**) Scheme of the experiment. IL-17A^eGFP^ × IFN-γ^Katushka^ × Foxp3^RFP^ triple reporter mice were injected DMSO or trans-Ned 19 2 h before first dose of anti-CD3 mAb. At 48 h, a second dose of anti-CD3 mAb was administered. The mice were sacrificed 4 h after second dose of anti-CD3 mAb. (**B**) Percentage of initial weight. *p*-Value was calculated with 2-way ANOVA. * *p* < 0.05.

In the next step, we assessed the therapeutic potential of NAADP inhibition of trans-Ned 19 in the context of intestinal inflammation. Thus, we administered it in vivo in the anti-CD3 mAb induced intestinal inflammation model in the triple reporter mice (IL-17A^eGFP^ × IFN-γ^Katushka^ × Foxp3^RFP^). This model is characterized by a cytokine storm and a transient intestinal inflammation accompanied by differentiation of Th17 to Tr1 cells [22,26,36]. Administration of trans-Ned 19 limited the extent of inflammation in this model. The mice treated with trans-Ned 19 had less profound systemic disease as indicated by lower extent of weight loss (Figure 7A,B).

## 4. Discussion

NAADP has been proven to play a pivotal role in TCR induced Ca^2+^ signaling and activation of T cells [17,18]. Moreover, progress has recently been made regarding the nature of a cytoplasmic receptor of NAADP, namely HN1L/JPT2, which acts as a NAADP binding protein and facilitates NAADP induced Ca^2+^ release by RYR1 or TPC1 [15,16]. Nevertheless, the role of NAADP signaling in CD4^+^ T-cell differentiation and its therapeutic potential in intestinal inflammation has not yet been studied.

Pharmacologic inhibition of NAADP signaling in primary murine and rat T cells has been previously shown to impair TCR dependent Ca^2+^ signaling and activation events [17,18]. In our study, we supplement these results by finding that trans-Ned 19 induced T-cell proliferation in response to TCR/CD3 stimulation can be reversed with protein kinase C activator PMA but not with the Ca^2+^ ionophore ionomycin. This surprising result points at the nonredundant role of NAADP in T-cell activation and might give a hint regarding NAADP synthesis pathway. The identity of NAADP synthesizing enzyme in vivo remains unknown. NAADP can be synthesized by purified CD38 in vitro [37,38]. However, gene silencing of *Cd38* in Jurkat T cells or its knockout in mice increased cytosolic levels of NAADP, suggesting rather a role of CD38 as NAADP degrading enzyme in vivo [39]. Billington et al. reported that NAADP can be reduced to NAADPH and suggested that NAADPH might act as an inert pool of NAADP [40]. In this study, NAADP was reduced to NAADPH by glucose-6-phosphatase dehydrogenase, an enzyme previously known to reduce NADP to NADPH [40]. By analogy, it can be hypothesized that NAADPH is oxidized to NAADP by NADPH oxidase (NOX). Isoforms of its catalytic component include NOX1, NOX2, NOX3, NOX4 and dual oxidases DUOX1 and DUOX2 [41]. Interestingly, DUOX1 can be activated by protein kinase A and DUOX2 by protein kinase C [42]. Thus, we can speculate that activation of protein kinase C activates DUOX2 enzyme, which synthesizes NAADP to overcome the trans-Ned 19 inhibition of T cell proliferation.

Interestingly, elevation of global [Ca^2+^] by ionomycin did not reverse the inhibition of TCR/CD3 induced proliferation by trans-Ned 19. This result points at the unique and non-redundant role of NAADP in TCR dependent signaling. Analogous findings regarding NAADP signaling in TCR downstream events was reported by Davis et al. [43]. In this study, NAADP stimulation of cytotoxic T cells evoked exocytosis which could be replicated by stimulating the cells with ionomycin and PMA but not with ionomycin alone.

Taken together, the results highlight the relevance of well-coordinated spatiotemporal patterns of NAADP induced Ca^2+^ microdomains in regulating T-cell receptor signaling.

We show that inhibition of NAADP signaling by means of trans-Ned 19 significantly interferes with the upregulation of TCR specific activation marker Nur77 and decreases the induction of CD69 and IRF4 expression. Upregulation of CD69 and IRF4 markers has been previously shown to be Ca^2+^ dependent and to control the metabolic function of the cell [30,43,44,45]. Consequently, trans-Ned 19 inhibited TCR induced metabolic reprogramming of naïve CD4^+^ T cells, as both oxygen consumption rate and glycolysis were decreased with the increasing doses of trans-Ned 19. Nevertheless, the significant changes were observed at the highest concentration of trans-Ned 19 used, which also completely blocked T-cell proliferation. The incomplete blockade of NAADP signaling using lower doses of the trans-Ned 19 did not result in significant inhibition of these activation events. In our study, we therefore explored the possible fine tuning of the T-cell differentiation by an intermediate antagonism of NAADP.

Interestingly, we show that partial inhibition of NAADP promoted the differentiation of Th1 and Th17 CD4^+^ T helper cells. Previous reports linking Ca^2+^ signaling and T-cell differentiation indicated that loss of function mutations impairing TCR induced Ca^2+^ signaling resulted in reduced production of cytokines by CD4^+^ T cells. For example, knockout of STIM1 and STIM2 or ORAI1, key molecules controlling store operated Ca^2+^ entry, impaired the differentiation of Th1 and Th17 cells in vitro and in vivo, and protected mice from experimental autoimmune encephalitis [3,4]. Moreover, another study has revealed that Ca^2+^ entry is critical for the pathogenicity of Th17 cells by promoting their metabolic activity [5]. Furthermore, Ali et al. reported that NAADP inhibition prevents the production of proinflammatory cytokines by T cells [18]. These results were obtained either with KO mice or with antagonists used at high concentration, thereby completely blocking the signaling via this pathway. In our study, we focused on the effects of partial inhibition of NAADP pathway which is also more likely to play a role in a therapeutic setting.

Moreover, we have shown that although NAADP inhibition by means of trans-Ned 19 promoted the differentiation of Th1 and Th17 T cells and impaired the differentiation of T regulatory cells, it also promoted production of IL-10 by these cells and co-expression of co-inhibitory receptors CD49b and LAG-3. Thus, the quality of these subsets changed to be more suppressive. IL-10 is a key anti-inflammatory cytokine which plays an essential role in the tolerance maintenance at the intestinal mucosal surfaces [46]. For instance, it has been shown that mice lacking IL-10 develop spontaneous colitis and that pro-inflammatory Th17 cells can be directly controlled by IL-10 [36]. Furthermore, Brockmann et al. reported that co-expression of co-inhibitory receptors identifies the highly suppressive IL-10 producing cells [34]. Thus, although the inhibition of NAADP signaling with trans-Ned 19 promoted the differentiation of IL-17A producing cells considered to be pro inflammatory effector cells, these cells had characteristics of regulatory cells and were suppressive in vitro.

Th17 cells can transdifferentiate into IL-10 expressing cells and limit the extent of inflammation [22]. We investigated this possibility and confirmed that NAADP signaling stabilizes the proinflammatory phenotype of Th17 cells as trans-Ned 19 promoted the differentiation of exTh17 Tr1 cells in vitro and in vivo. We evaluated the therapeutic utility of NAADP inhibition in the preclinical model of anti-CD3 induced intestinal inflammation [26]. Inhibition of NAADP ameliorated the course of disease in this model.

Taken together, we present a novel mechanism explaining how NAADP modulates T cells responses by impacting the transdifferentiation of Th17 cells into regulatory T cells. This finding builds the basis for future targeted therapies aiming to control Th17 cell mediated inflammatory diseases.

## Data Availability

The data presented in this study is contained within the article or supplementary material.

## Supplementary Materials

The following are available online at https://www.mdpi.com/article/10.3390/cells10113039/s1, Figure S1: Gating strategy used in flow cytometry experiment with trans-Ned 19. Figure S2: NAADP inhibition by trans-Ned 19 does not decrease the viability of resting CD4^+^ T cells in vitro. Figure S3: NAADP inhibition by trans-Ned 19 promotes the differentiation of Th1 and Th17 cells upon stimulation with antigen. Figure S4: CD4^+^ T cell in vitro differentiation in the presence of NAADP antagonist BZ194.
