# Supplementary material for: Trans-Ned 19-Mediated Antagonism of Nicotinic Acid Adenine Nucleotide—Mediated Calcium Signaling Regulates Th17 Cell Plasticity in Mice
Source: Cells. 2021 Nov 5;10(11):3039. doi: 10.3390/cells10113039 (PMC8616272; doi:10.3390/cells10113039)
Supplement: Supplementary file 1 [file cells-10-03039-s001.zip › cells-1410402-supplementary.pdf]

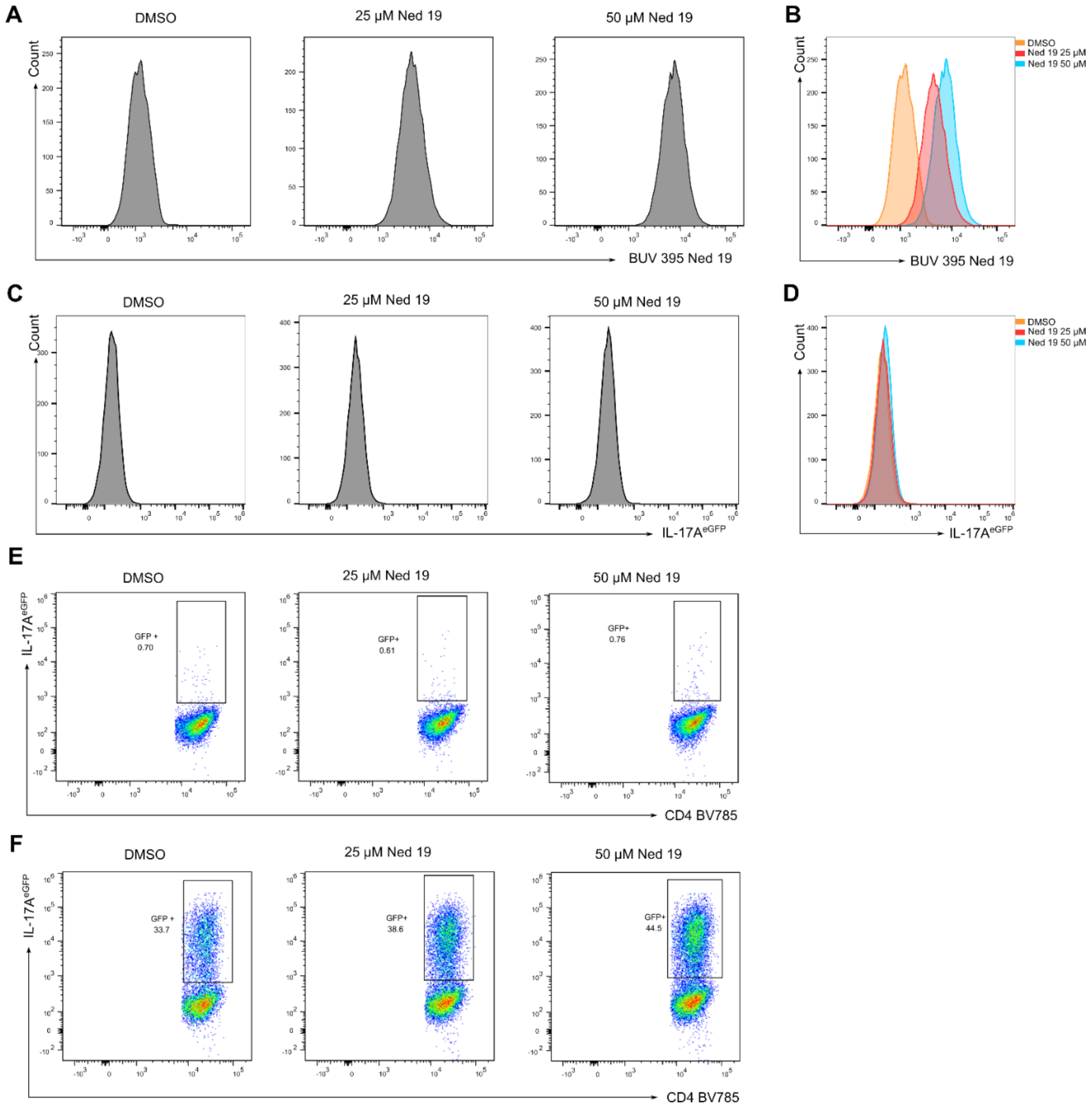

Supplementary Figure S1. Gating strategy used in flow cytometry experiment with trans-Ned 19. Naïve $CD4^+$ T cells were isolated from and IL-17A^{eGFP} × IFN-γ^{Katushka} × Foxp3^{RFP} reporter mice. Cells were incubated with DMSO or trans-Ned 19 and differentiated in vitro. (**A**) Mean fluorescence intensity in BUV 395 channel. (**B**) Overlay of the MFI histograms in BUV395 channels. (**C**) Mean fluorescence intensity in GFP channel. (**D**) overlay of the MFI histograms in GFP channels. (**E**) Gating of GFP^+ events using GFP-negative condition. (**F**) Gating of GFP^+ events in Th17 condition.

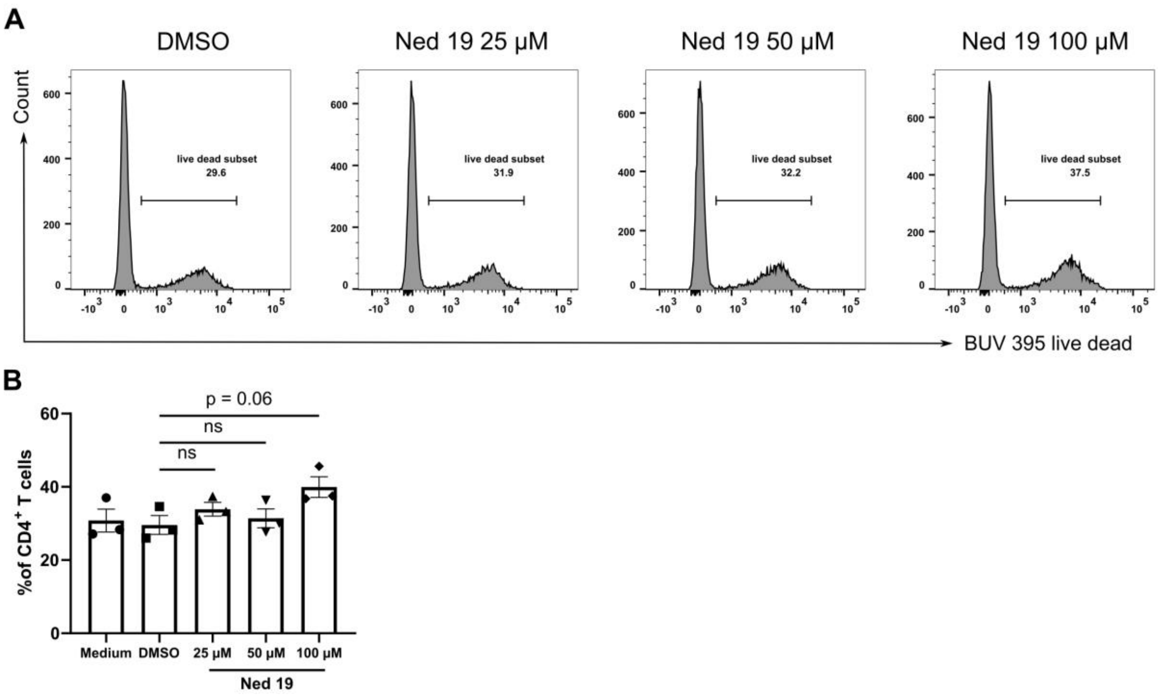

Supplementary Figure S2. NAADP inhibition by trans-Ned 19 does not decrease the viability of resting CD4+ T cells *in vitro*. The CD4+ T cells were freshly isolated from WT C57BL/6N mice, incubated with increasing concentrations of trans-Ned 19 for 1 h and cultured for 24 h in presence of IL-7 (10 ng/mL). (**A**) Representative histograms of live dead staining. (**B**) Summary statistics of viable cells. Presented data in B are mean ± SEM. The p-values were calculated with repeated measures ANOVA with Dunnett's multiple comparison test.

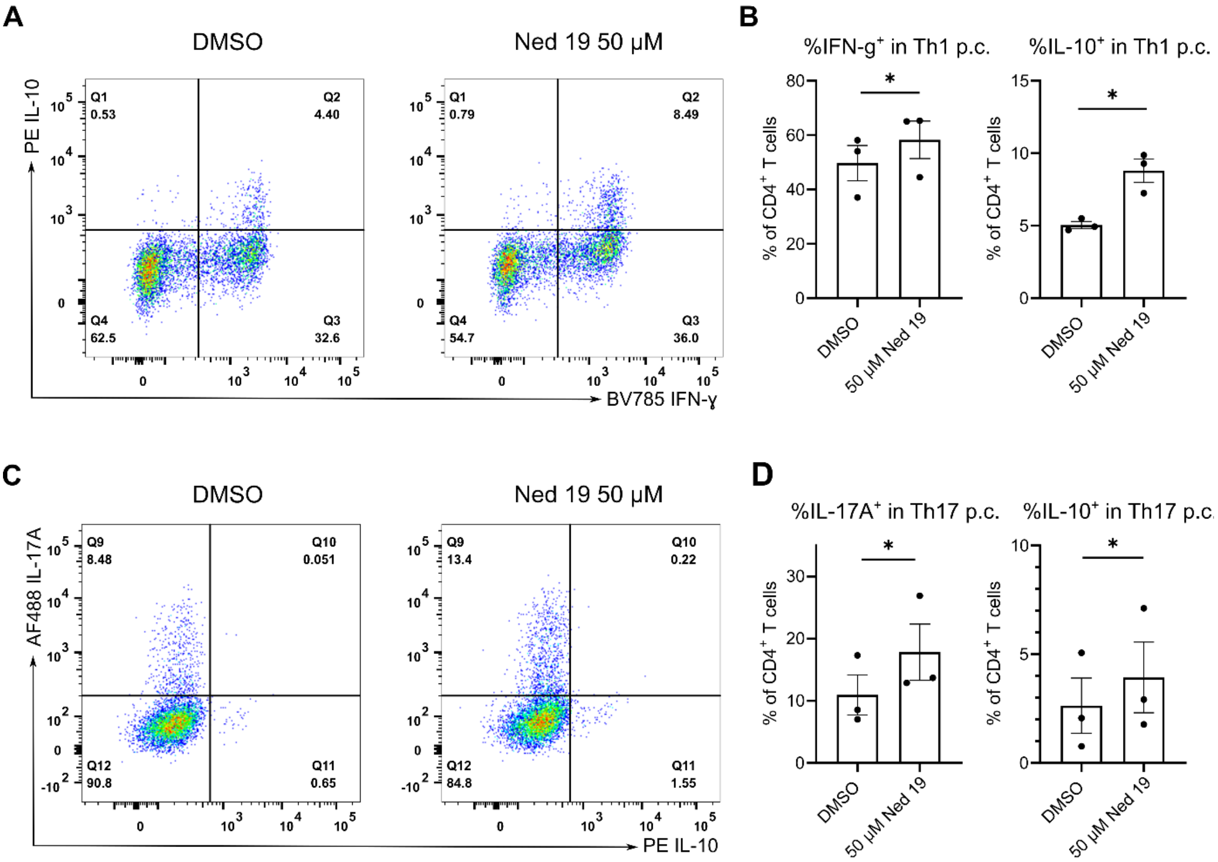

Supplementary Figure S3. NAADP inhibition by trans-Ned 19 promotes the differentiation of Th1 and Th17 cells upon stimulation with antigen. The naïve CD4+ T cells were isolated from OT-II mice and incubated with increasing concentrations of trans-Ned 19 for 1 h. Cells were stimulated with ovalbumin peptide and CD3-

depleted splenocytes under Th1 and Th17 polarizing conditions in presence of trans-Ned 19. (**A**) Representative staining for IFN-γ and IL-10 after differentiation under Th1 polarizing conditions. (**B**) Summary statistics of IFN-γ and IL-10 expression under Th1 polarizing conditions (p.c.). (**C**) Representative staining for IL-17A and IL-10 after differentiation under Th17 polarizing conditions. (**D**) Summary statistics of IL-17A and IL-10 expression under Th17 polarizing conditions. The data points in (**B**) and (**D**) are total percentages of IFN-g$^+$, IL-17A$^+$ and IL-10$^+$ of CD4$^+$ T cells. The p-values were calculated with paired Wilcoxon test. * $p < 0.05$.

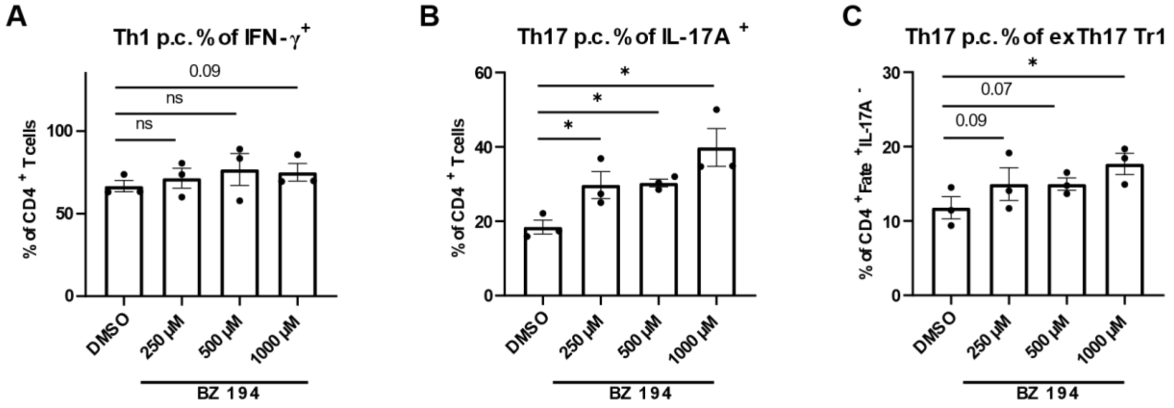

Supplementary Figure S4. CD4$^+$ T cell *in vitro* differentiation in the presence of NAADP antagonist BZ194. Naïve CD4$^+$ T cells were freshly isolated from IL-17A^{eGFP} × IFN-γ^{Katushka} × Foxp3^{RFP} mice. Cells were incubated with increasing concentrations of BZ194 for 5 h and stimulated with plate bound anti-CD3 mAb, soluble anti-CD28 mAb and polarizing cytokine cocktails. (**A**) Summary statistics of Th1-cell differentiation (p.c. polarizing conditions). (**B**) Summary statistics of Th17-cell differentiation (**C**) Summary statistics of Treg differentiation. (**D**) Naive CD4$^+$ T cells were freshly isolated from Fate reporter mice (IL-17A^{Cre} × Rosa26 STOP$^{fl/fl}$ YFP × 17A^{Katushka} × IL-10^{eGFP} × Foxp3^{RFP} mice). Cells were incubated with increasing concentrations of BZ194 for 5 h, stimulated with plate bound anti-CD3 mAb, soluble anti-CD28 mAb and cultured for 96 h in the presence of BZ194 under Th17 polarizing conditions. Mean ± SEM are depicted on the graphs. P-values were calculated with repeated measures ANOVA with Dunnet's multiple comparison test. * $p < 0.05$.
